# UNDERSTANDING SEVERITY OF ELDER MISTREATMENT: FINDINGS FROM THE CANADIAN LONGITUDINAL STUDY ON AGING

**DOI:** 10.1093/geroni/igae098.2063

**Published:** 2024-12-31

**Authors:** David Burnes, Mark Lachs, Karl Pillemer

**Affiliations:** University of Toronto, Toronto, Ontario, Canada; Weill Cornell Medicine, New York, New York, United States; Cornell University, Ithaca, New York, United States

## Abstract

Basic elder mistreatment knowledge on risk factors and prevalence has advanced substantially over the past two decades. However, research in this area has predominantly framed our understanding of elder mistreatment in binary (no/yes) terms. While a binary understanding of elder mistreatment is necessary to determine prevalence/incidence, this conventional framing obscures the variation in lived experiences across cases and provides limited clinical insights for response interventions seeking to reduce harm. Building on prior research examining elder mistreatment through a lens of severity, this study analyzed data from the population-based Canadian Longitudinal Study on Aging to both describe the range of elder mistreatment severity across cases and identify longitudinal risk/protective factors associated with severity levels. An ecological-systems perspective organized potential risk/protective factors from several levels of influence. Drawing on a nationally stratified, random sample (n=23,468) of community-dwelling adults aged 65 or older interviewed over a three-year period, the current study analyzed a subsample (n=2368) of older adults identifying as victims of emotional, physical, or financial mistreatment. Severity was operationalized according to past-year frequency and multiplicity of mistreatment behaviors experienced. Ordinal/multinomial logistic regression was used to examine static and change risk/protective factors for elder mistreatment in general and for separate subtypes. Older adults with lower physical, cognitive and mental health, higher levels of child maltreatment, who lived with their perpetrator, and identified as female were more likely to experience severe forms of mistreatment. Findings from this study advance our basic knowledge on elder mistreatment phenomena and inform practice in community-based response programs.

